# Dihydroceramide Desaturase Inhibition by a Cyclopropanated Dihydroceramide Analog in Cultured Keratinocytes

**DOI:** 10.1155/2011/724015

**Published:** 2010-12-05

**Authors:** Susanne Brodesser, Thomas Kolter

**Affiliations:** ^1^CECAD Cologne Platform Lipidomics, Institute for Medical Microbiology, Immunology and Hygiene, University of Cologne, Goldenfelsstraße 19-21, 50935 Cologne, Germany; ^2^LIMES Program Unit Membrane Biology and Lipid Biochemistry, Kekulé Institute of Organic Chemistry and Biochemistry, University of Bonn, Gerhard-Domagk-Straße 1, 53121 Bonn, Germany

## Abstract

Most mammalian sphingolipids contain a 4,5-(*E*)-double bond. We report on the chemical synthesis of a dihydroceramide derivative that prevents the introduction of the double bond into sphingolipids. Minimal alteration of the parent structure by formally replacing the hydrogen atoms in the 5- and in the 6-position of the sphinganine backbone by a methylene group leads to an inhibitor of dihydroceramide desaturase in cultured cells. In the presence of 10–50 *μ*M of compound (**1**), levels of biosynthetically formed dihydroceramide and—surprisingly—also of phytoceramide are elevated at the expense of ceramide. The cells respond to the lack of unsaturated sphingolipids by an elevation of mRNAs of enzymes required for sphingosine formation. At the same time, the analysis of proliferation and differentiation markers indicates that the sphingolipid double bond is required to keep the cells in a differentiated state.

## 1. Introduction

**Scheme 1:**
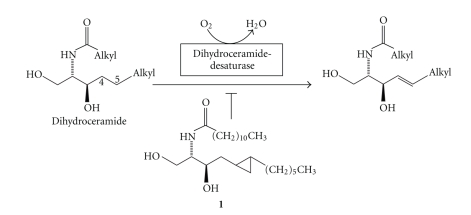
Enzymatic reaction catalyzed by dihydroceramide desaturase DES1.

Most mammalian sphingolipids contain an (*E*)*-*configured double bond between the carbon atoms C4 and C5 of the sphingoid moiety [1–3]. This structural element is introduced during the last step of ceramide biosynthesis by the enzyme dihydroceramide desaturase (DES1; Scheme 1), a protein bound to the membrane of the Endoplasmic Reticulum [4–7], encoded by the DEGS1 (degenerative spermatocyte homolog 1) gene, and myristoylated at its *N*-terminus [8]. While the mouse Des2 protein seems to have dihydroceramide Δ4-desaturase and 4-hydroxylase activity, the human homolog DES2 appears to be only a 4-hydroxylase [9]. The first and rate-determining step of the dihydroceramide desaturase-catalyzed reaction is the homolytic cleavage of the C-H-bond at C-4 of the sphinganine backbone by a non-heme oxo-diiron species [10]. The function of the double bond within the sphingolipids is not entirely known. Experiments, in which cell-permeable analogs of ceramide and dihydroceramide were exogenously added to cultured cells [11], indicated for the first time that the double bond is not only required for the signaling properties of ceramide, but also for those of sphingosine-1-phosphate (review: [12]). Dihydroceramide desaturase (Des1) knockout mice display decreased body weight, scaly skin, hematological abnormalities, and abnormal liver function, and die at 8 to 10 weeks after birth [13]. As an additional tool to study the function of the 4,5-double bond of sphingolipids, competitive inhibitors of the desaturase have been developed that share a cyclopropene moiety in positions 4 and 5 of the sphingoid backbone [14–18]. Dihydroceramide levels also increase in response to resveratrol treatment [19].

## 2. Results and Discussion

Here, we report on the synthesis of a cyclopropane-bearing dihydroceramide derivative that is able to inhibit the introduction of the double bond into the sphingolipids of cultured cells. The dihydroceramide analog (**1**) (Scheme 1) is structurally very closely related to dihydroceramide and distinguished from it only by the presence of an additional methylene group. In addition, the acyl chain is shorter than that of most endogenous ceramides to ensure sufficient cell permeability. Since exogenously added short-chain ceramides are converted in cells into those with endogenous acyl chain lengths by deacylation/reacylation [20], the title compound can behave as the inhibitor or also as the prodrug of a cyclopropanated dihydroceramide of native acyl chain length. In vitro, the desaturase shows optimal activity towards substrates with acyl chain lengths of eight carbon atoms [4], and also inhibition by the cyclopropene-based inhibitor is optimal with this chain length [15]. However, nothing is known about the chain length that is actually optimal in cells. The rationale behind the synthesis of the title compound was that enzyme-catalyzed homolytic cleavage of the C4-H bond within the sphingoid scaffold of **1** and subsequent opening of the cyclopropyl ring should lead to a delocalized radical intermediate that might covalently bind to and in this way inactivate the desaturase. Mechanism-based inhibition of oxidizing enzymes by cyclopropyl-containing substrate analogs has been described before, for example, [21].

**Scheme 2:**
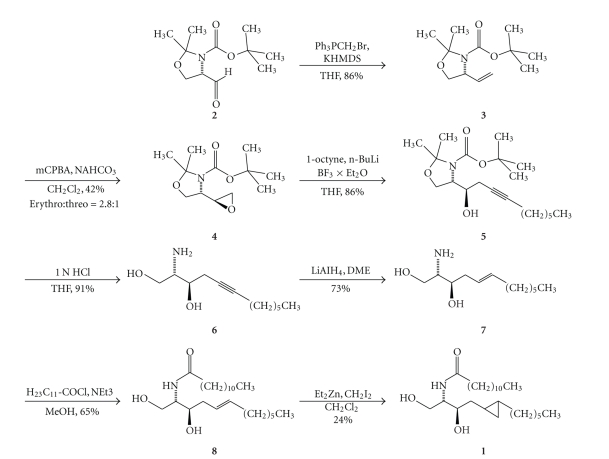
Reagents and conditions: (a) Ph_3_PCH_3_Br, KHMDS, THF, −78°C then rt, 2 h (86%); (b) mCPBA, 0.5 M NaHCO_3_, CH_2_Cl_2_, 0°C then rt, 18 h (42%, *erythro:threo* = 2.8 : 1); (c) 1-octyne, n-BuLi, BF_3_ × Et_2_O, THF, −78°C, 30 min (86%); (d) 1 N HCl, THF, 70°C, 18 h (91%); (e) LiAlH_4_, DME, 90°C, 12 h (73%); (f) H_23_C_11_COCl, NEt_3_, MeOH, 0°C then rt, 18 h (65%); (g) Et_2_Zn, CH_2_I_2_, CH_2_Cl_2_, rt, 24 h (24%).

The synthesis of **1** (Scheme 2) started from Garner's serine aldehyde (**2**) [22], which was transformed into **3** by a Wittig olefination. Treatment of **3** with *meta*-chloroperbenzoic acid led to a 2.8 : 1 diastereomeric mixture of *erythro*- and *threo*-epoxides. Pure *erythro*-epoxide (**4**) was isolated after flash chromatography and elongated with lithiated octyne to **5**.

**Scheme 3:**
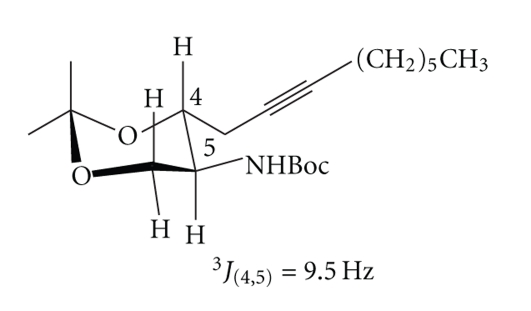


The relative configuration of the carbon atoms C2 and C3 of *erythro*-**5 **was determined after selective cleavage of the acetal moiety with *Amberlyst 15 *in methanol at room temperature and subsequent formation of the 1,3-isopropylidene acetal [23]. ^1^HNMR analysis showed a coupling constant ^3^J_(4,5)_ of 9.5 Hz, which confirmed the *trans*-diaxial relationship of H-4 and H-5 (Scheme 3). The *threo-*diastereomer had a ^3^J_(4,5)_ coupling constant of about 1 Hz.

After acid deprotection of *erythro*-**5** (Scheme 2), the homopropargylic alcohol (**6**) was reduced to the homoallylalcohol (**7**) with lithium aluminium hydride. Acylation with lauroyl chloride afforded **8**. Subsequent introduction of the cyclopropane moiety by the Furukawa variant of the Simmons-Smith reaction [24] led to two diastereomers of **1**, which could not be separated.

**Figure 1:**
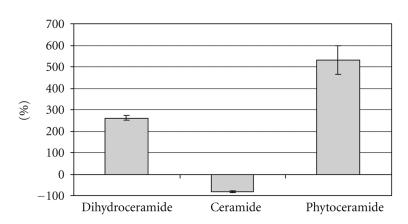
Incorporation of l-[3-^14^C]serine into ceramides of differentiated keratinocytes in the presence of 50 *μ*M of **1**. Deviations [%] from levels obtained in untreated cells, which were cultured as previously described [29], are shown.

Since (dihydro)ceramide analogs exogenously added to cultured cells undergo similar metabolic reactions as endogenous ceramides [25], the rapid conversion of **1** by ceramide metabolizing enzymes and termination of its effect on the desaturase system had to be expected. Compared to other tissues, the human skin is particularly rich in ceramides [26–28]. Therefore, in this cell type, sufficiently high steady-state-concentrations of **1** or of a derivative of **1** generated by de- and reacylation with endogenous fatty acids could be expected to prevent the introduction of the double bond. Moreover, a cell culture model of the human skin is available and allows the analysis of markers of differentiation and proliferation, which should be affected by the presence of sphingolipids with and without 4,5-(*E*)-double bond. For these reasons we investigated **1** in cultured differentiated human keratinocytes [29]. After seven days of differentiation, the cells were incubated with different concentrations of **1** in the culture medium for 24 hours. The differentiated keratinocytes were then metabolically labeled with l-[3-^14^C] serine, the biosynthetic precursor of sphingolipids, for 72 hours. Lipids were extracted, separated by *thin layer chromatography* on silica gel plates impregnated with sodium borate [30], visualized by phosphoimager analysis, and quantified (Figure 1).

The analysis revealed a concentration-dependent decrease of l-[3-^14^C]serine incorporation into ceramide. 50 *μ*M concentration of **1** in the culture medium led to a reduction of ceramide labeling to less than 20% of untreated cells. Dihydroceramide labeling was increased 3.6-fold, and labeling of phytoceramide, which is formed by 4-hydroxylation of dihydroceramide (the accumulated desaturase substrate), was 6.3-fold elevated compared to untreated cells. In contrast to the title compound **1**, the cyclopropene-containing inhibitor GT11 [14] did not lead to an elevation of phytoceramide labeling (Bernadette Breiden, personal communication). The effect of **1** on dihydroceramide and ceramide biosynthesis as determined by l-3-[^14^C]serine labeling was confirmed with *N*-[1-^14^C]octanoyl-sphinganine, which is a cell-permeable analog of dihydroceramide. Also labeling of the ceramide subspecies Cer(AS), which consists of sphingosine acylated with a 2-hydroxy-fatty acid, was reduced. Moreover, labeling of ceramides composed of 2-hydroxyfatty acids and hydroxylated sphingoid bases (Cer(AP) and Cer(AH)) was enhanced in the presence of **1** (data not shown). In agreement with the proposed mechanism of action of the title compound, corresponding dihydroceramides with cyclopropane moieties between C4 and C5, as well as between C6 and C7 of the sphinganine backbone, were inactive (unpublished), although competitive inhibition had to be expected.

Since desaturation and hydroxylation of dihydroceramide should follow a similar mechanism, it is remarkable that **1** leads to an accumulation of dihydroceramide and phytoceramide on the expense of ceramide levels. The keratinocyte-specific disruption of the gene encoding the transcription factor *Arnt *in mice led to severe transepidermal water loss because of low levels of both, ceramides and phytoceramides, and elevated concentrations of dihydroceramide in the animals' epidermis [31]. Enzymes with both sphingolipid 4-desaturase and 4-hydroxylase activity have been suggested to exist in mice [32, 33] and *Candida albicans *[34]. Furthermore, a human homolog of the mouse 4-desaturase/4-hydroxylase has been described. Its mRNA expression was shown to be induced during ascorbate/serum-induced differentiation of human keratinocytes [35].

**Figure 2:**
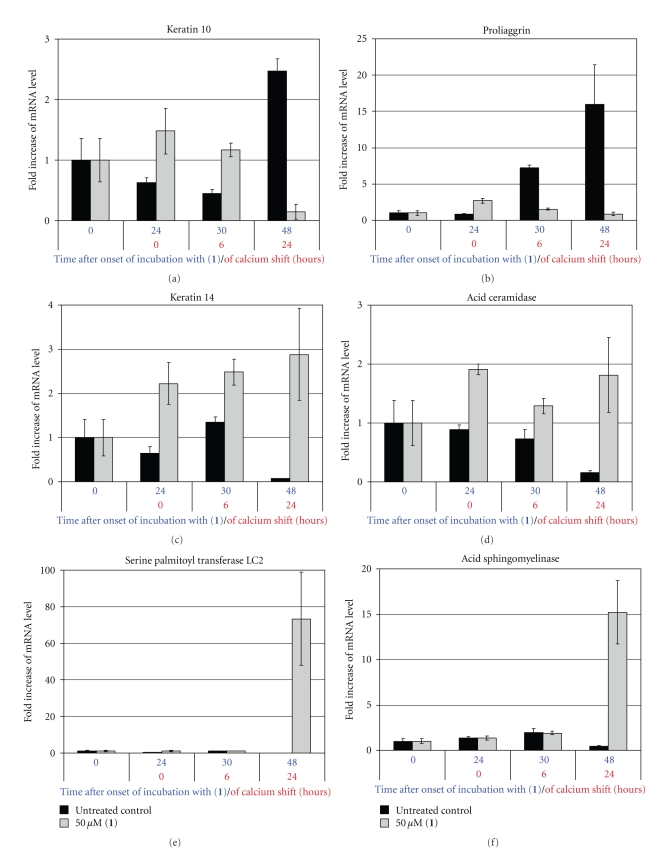
Effect of **1** on transcription levels of differentiation markers and enzymes of sphingolipid metabolism in cultured human keratinocytes. Proliferating cells were incubated with 50 *μ*M of **1** for 24 h and were then submitted to a *calcium shift* to induce differentiation.

In order to investigate functional consequences of the reduction of newly biosynthesized unsaturated sphingolipids brought about by **1**, we quantified the mRNA levels of several proteins involved in cell differentiation and ceramide metabolism by *real-time polymerase chain reaction* (Figure 2).

For this purpose, proliferating human keratinocytes were preincubated with **1 **for 24 hours. Differentiation was then induced by submitting the cells to an increased calcium ion concentration in the medium, the so-called *calcium shift *[29]. Former studies already demonstrated a correlation between mRNA and protein levels under these conditions [36].

Figure 2 shows that 24 hours after the onset of the *calcium shift*, the transcription levels of the suprabasal differentiation marker genes [37], keratin 10 and profilaggrin, were about 95% lower in cells treated with **1** than in untreated cells at the same point of time. On the other hand, the transcription level of the basal marker gene [36], keratin 14, was 40-fold elevated. These expression patterns suggest that treatment with **1** causes an impairment of differentiation, so that the cells remain in a basal proliferative state. The most likely explanation is to attribute this effect to the lack of newly synthesized sphingolipids with 4,5-double bond.

We also determined the mRNA expression of several enzymes involved in ceramide metabolism (Figure 2). The mRNA level of subunit 2 of serine palmitoyl transferase, the enzyme catalyzing the rate-determining step of sphingolipid *de novo* synthesis, increased more than 70-fold within 48 hours after the onset of incubation with **1**, whereas the expression in untreated keratinocytes remained low. Also, the transcription levels of glucosylceramide-*β*-glucosidase (not shown) and of acid sphingomyelinase, which produce ceramide by the breakdown of glucosylceramide and sphingomyelin, respectively, were upregulated by **1** compared to control cells. Unexpectedly, the cells respond to an exogenously added excess of a ceramide analog with an upregulation of proteins required for ceramide formation. However, also the transcription of the gene encoding acid ceramidase that cleaves ceramide into sphingosine and fatty acid was upregulated. These results suggest that increased concentrations of saturated sphingolipids direct sphingolipid metabolism to the production of sphingosine, which can then be phosphorylated to sphingosine-1-phosphate. In contrast to ceramide, sphingosine-1-phosphate behaves as a stimulator of proliferation in most cell types [12]. This again indicates that the 4,5-double bond is required for keratinocyte differentiation, which is disturbed by the accumulation of ceramides saturated within the sphingoid moiety.

**Figure 3:**
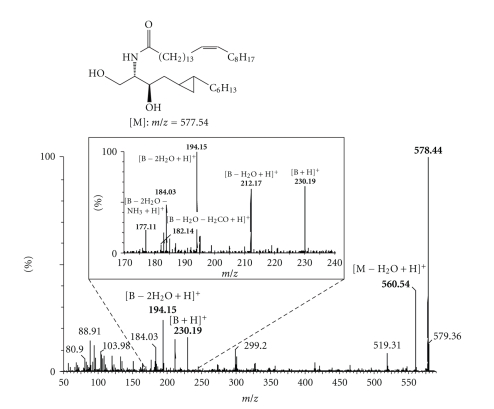
MS/MS spectrum of the metabolite of **1** acylated with nervonic acid (B = sphingoid base).

Furthermore, we identified several metabolites of **1** produced by ceramide metabolizing enzymes within the cell. The analysis was done by *electrospray ionization mass spectrometry* (ESI-MS) in lipid extracts of differentiated keratinocytes treated with **1**. In this way, *N*-acyl metabolites of **1**, produced by ceramidase-catalyzed cleavage of the amide bond and subsequent acylation with different endogenous fatty acids, were found (Table 1). As an example, Figure 3 shows the MS/MS spectrum of the metabolite of **1** acylated by nervonic acid. In addition, the sphingomyelin analog of **1**, but not the corresponding glucosylated metabolite, could also be identified.

In summary, we demonstrated that addition of nontoxic concentrations of the dihydroceramide analogue **1** to cultured keratinocytes reduces the introduction of the double bond into newly synthesized sphingolipids and leads to an impairment of differentiation. As a result, the keratinocytes remain in a basal proliferative state. This indicates that the double bond within the sphingoid moiety of sphingolipids is important for the maintenance of differentiation. 

Despite the apparent similarity between the title compound and the family of cyclopropene-based dihydroceramide desaturase inhibitors, there are principle differences between both approaches: cyclopropenes display significant chemical reactivity towards nucleophiles and behave as alkylating agents. Cyclopropene-containing fatty acid analogs interfere with desaturation by addition to thiol groups of cysteine residues [38]. Alkyl cyclopropanes, on the other hand, require oxidation or hydrogen abstraction in *α*-position for reactivity. The cyclopropene inhibitors [14–17] are potent and selective dihydroceramide desaturase inhibitors and have been applied, for example, to analyze the function of dihydrosphingolipids [39]. The best characterized parent compound is a competitive inhibitor with a *K*_*i*_ value of 6 *μ*M [14]. Side effects, which occur at concentrations above 5 *μ*M, such as inhibition of serine palmitoyl transferase and accumulation of sphingoid-1-phosphates, have been investigated in neurons [18]. We add information on the new title compound and demonstrate its effect on differentiation and proliferation of cultured human keratinocytes. Since low-molecular weight sphingolipid analogs are discussed as tools for the control of signaling and disease [40], the title compound with only a minimal change in the dihydroceramide structure might be of value for the controlled alteration of cellular properties.

## 3. Experimental

### 3.1. Analytical Data

*N*-((1*S*,2*R*)-(3-(2′-Hexylcyclopropyl)-2-hydroxy-1-hydroymethyl-propyl)) dodecanamide (**1**): R_f_ (chloroform/methanol 20 : 1) = 0.23.

^1^H-NMR (300 MHz, CDCl_3_): *δ*  =  0.15 − 0.31 (m, br, 2H, cyclopropyl-H), 0.36 − 0.55 (m, br, 2H, cyclopropyl-H), 0.86 (t, J  =  6.5 Hz, 6H, CH_3_), 1.13 − 1.41 (m, 28H, CH_2_), 1.41 − 1.50 (m, br, 1H, cyclopropyl-H), 1.62 (m, 2H, CH_2_), 2.21 (t, J = 7.5 Hz, 2H, CH_2_CO), 2.50 − 2.85 (m, br, 2H, *H*OCH_2_, 2-OH), 3.69 (dd, J = 11.0 Hz, J = 3.0 Hz, 1H, HOCH_A_H_B_), 3.81 − 3.90 (m, br, 2H, 1-H, 2-H), 3.95 (dd, J = 11.0 Hz, J = 3.0 Hz, 1H, HOCH_A_H_B_), 6.36 (d, J = 7.0 Hz, 1H, NH). ^13^C-NMR (75 MHz, CDCl_3_): *δ*  =  11.2 (11.9) (cyclopropyl-C), 14.1 (CH_3_), 15.1 (15.2) (cyclopropyl-C), 18.1 (19.1) (cyclopropyl-C), 22.7 − 34.0 (CH_2_), 36.9 (CH_2 _CO), 38.9 (3-C), 53.6 (1-C), 62.4 (HOCH_2_), 74.8 (2-C), 173.6 (CO). The signal duplication for the carbon atoms of the cyclopropane ring in 5,6-position of the dihydroceramide scaffold can be attributed to the formation of two diastereomers with different configuration during the Simmons-Smith reaction.

FAB-MS (mNBA, C_25_H_49_NO_3_): *m*/*z*  =  412 [M + H]^+^, 450 [M + K]^+^; EI-MS (HR-MS): calcd.: 411,3712, found: 411,3711.

Cell culture experiments and quantification of mRNA levels via real-time quantitative PCR were performed according to [36]. 

### 3.2. Cell Culture

Human foreskin keratinocytes were cultivated according to a modified Rheinwald and Green method [36] and grown to confluence in low calcium (0.1 mM) MCDB-153 medium. Differentiation was initiated by elevating the calcium ion concentrations to 1.1 mM (“calcium shift”) and addition of linoleic acid/BSA (10 *μ*M) to the MCDB-153 medium. In the presence of inhibitor **1**, cells were viable as determined by morphology, cell protein content, and metabolic capability to incorporate radiolabeled serine into sphingolipids, when compared to untreated cells.

### 3.3. Ceramide Labeling

After 8 days of calcium shift, the medium was replaced by the corresponding serine-deficient medium and l-[3-^14^C]serine was added (1 *μ*Ci/mL medium) after one hour. Cells were harvested 72 hours after the addition of radiolabeled serine. **1** was dissolved in ethanol (10 mM solution) and added to the cell culture medium 24 hours before labeling. All cell culture experiments were conducted at least in duplicate.

### 3.4. Lipid Extraction and Analysis

Cells were harvested and then homogenized by sonication in water. Lipids were extracted by addition of methanol and chloroform and subsequent incubation at 37° for 24 h. Lipid extracts were desalted on LiChroprep RP-18 columns, and the incorporated radioactivity was quantified by liquid scintillation counting (Packard Instruments, Tri-Carb 1600 TR). In order to quantify the relative distribution of radioactivity within the different ceramide subspecies, TLC separation using the solvent systems chloroform/methanol 9 : 1 (v/v) on borate-impregnated silica gel plates and phosphoimager analysis (Fuji, Fujix BAS 1000) were performed. Ceramide (Cer(NS)), dihydroceramide (Cer(ND)), and phytoceramide (Cer(NP)) were clearly separated by this method. To separate ceramide subspecies, Cer(NS) and Cer(ND), from those acylated with 2-hydroxy-fatty acids (Cer(AS), Cer(AP), and Cer(AH)), TLCs were run twice in chloroform/methanol/acetic acid 190 : 9 : 1 (v/v/v). Lipids were identified by comparing their mobility with authentic reference standards.

### 3.5. Electrospray Ionization-Time of Flight-Mass Spectrometry

Measurements were conducted on a Q-ToF-2 Hybrid Quadrupole-mass spectrometer with nanoflow interface (Micromass) in positive or negative ion mode. Lipid extracts were evaporated in a stream of nitrogen, dissolved in Chloroform/Methanol 1 : 1 (v/v), and sonicated for five minutes. For the suppression of sodium adducts, the sample was mixed with an aqueous solution of 1 M ammonium acetate to a final concentration of 50 mM. The sample was vortexed and centrifuged. 5 *μ*L of the supernatant were applied to precoated glass capillaries and measured at a source temperature of 80°C, a capillary voltage of 900–1000 V, and a cone voltage of 50 V. Calibration was performed using sodium iodide (2 mg/mL) and cesium iodide (0.05 mg/mL) in 1-propanol/water 1 : 1 (v/v). MS/MS spectra were recorded with argon as collision gas at an argon partial pressure of 0.7 bar. Collision energies were in the range of 30 to 40 eV. 

### 3.6. RNA Extraction and Quantification

In order to investigate the effect of **1** on the differentiation of human keratinocytes, the cells were grown to confluence as described above. 24 hours before the onset of calcium shift, **1** was added to the cell culture for preincubation. The cells were harvested after the indicated periods of time (see Figure 2), and total RNA was extracted using the Purescript RNA isolation kit according to the supplier's instructions. RNA quality was tested using a 1.2% standard RNA agarose gel and by measuring the optical density ratio A_260_/A_280_ (≥2.0). RNA solutions were then diluted to 25 ng/*μ*l in sterile water treated with diethyl pyrocarbonate (DEPC) and stored at –80°C.

### 3.7. Real-Time PCR RNA Quantification

For every gene studied, primers were dissolved in DEPC-treated sterile water. All fluorogenic probes contained the reporter dye FAM covalently attached to the 5′-end and the quencher dye TAMRA covalently attached to the 3′-end. Extension from the 3′-end was blocked by attachment of a 3′-phosphate group.

### 3.8. cDNA Synthesis

The target RNA (75 ng) was reversetranscribed using the MuLV reverse transcriptase (Applied Biosystems) at 48°C for 30 min. The total reaction volume was 20 *μ*L. After cDNA synthesis, reverse transcriptase was denatured at 95°C for 10 min.

### 3.9. PCR Amplification

PCR reactions were performed with the ABI Prism 7700 sequence detection system (Applied Biosystems). 20 *μ*l of the RT-PCR reaction from each cDNA sample were amplified using the AmpliTaq Gold (Applied Biosystems) in a total volume of 90 *μ*l. Each amplification was performed in triplicate under the following conditions: 2 min at 50°C and 10 min at 95°C, followed by a total of 45 two-step cycles (15 s at 95°C and 1 min at 60°C). As an endogenous control, the porphobilinogen deaminase (PBGD) gene mRNA was used, which was quantified in parallel. The results were analyzed utilizing the standard curve method as described in User Bulletin number 2 (ABI Prism 7700 sequence detection system, Applied Biosytems).

## Figures and Tables

**Table 1:** *N*-acyl metabolites of **1** identified by ESI-MS in lipid extracts of differentiated keratinocytes.

Fatty acid type	*m*/*z* of molecular ion [M + H]^+^
14 : 0	440.4
16 : 0	468.4
16 : 0h^a^	484.4
18 : 0	496.4
18 : 1	494.4
22 : 0	552.4
24 : 1	578.5
26 : 0	608.5
28 : 1	634.5

^a^ *α*-hydroxy fatty acid.
